# Mixed-dimensional fluid–structure interaction simulations reveal key mechanisms of cerebrospinal fluid dynamics in the spinal canal

**DOI:** 10.1186/s12987-025-00691-4

**Published:** 2025-07-30

**Authors:** Deshik Reddy Putluru, Adrian Buganza Tepole, Hector Gomez

**Affiliations:** 1 School of Mechanical Engineering, Purdue University, 585 Purdue Mall, West Lafayette, 47906 IN USA; 2 Weldon School of Biomedical Engineering, Purdue University, 206 S Martin Jischke Dr, West Lafayette, 47906 IN USA

**Keywords:** Cerebrospinal fluid dynamics, Dura mater, Tissue mechanics, Fluid-structure interaction

## Abstract

Cerebrospinal flow dynamics (CSF) plays a critical role in structural disorders of the central nervous system (CNS) and in the design of effective procedures for intrathecal drug delivery. Medical imaging techniques have only partially characterized CSF dynamics. Computational models have the potential to offer a high-resolution description of CSF flow and advance our mechanistic understanding. However, anatomically-accurate computational models of CSF dynamics in the spinal canal have largely ignored the compliance of the spinal tissues, which is critical to understand the pulse wave velocity and the craniocaudal decay of CSF pulsations. Here, we propose a mixed-dimensional fluid-structure interaction method that enables high-fidelity simulations of CSF dynamics on anatomically-accurate models of the spinal canal, considering the tissue compliance effects emerging from the dura mater and epidural fat. Our mixed-dimensional approach bypasses a critical computational bottleneck that emerges from the multiscale geometry of spinal tissues. Our results show that accurate modeling of tissue compliance is critical to capture key elements of CSF dynamics. This work opens new possibilities to control and optimize intrathecal drug delivery and to understand structural abnormalities of the CNS.

**Supplementary Information:**

The online version contains supplementary material available at 10.1186/s12987-025-00691-4.

## Introduction

Central nervous system (CNS) disorders are a serious concern to global healthcare systems. Most of these conditions are incurable, and often require frequent therapeutic interventions to alleviate symptoms or slow down the disease progression [1, 2]. A major challenge in treating CNS disorders is drug delivery. The blood-brain barrier (BBB) blocks foreign agents from entering the CNS, including 98$\%$ of drug molecules [3]. Hence, conventional routes of drug administration such as intravenous, oral and subcutaneous are ineffective in CNS therapeutics. Intrathecal administration is a promising approach to deliver drugs to the CNS. In this delivery modality, the therapeutic agent is injected in the spinal subarachnoid space (SAS), the region of the CNS that is located between the dura mater and the spinal cord. The SAS is filled with cerebrospinal fluid (CSF), which flows around the brain and the spinal cord and acts as a carrier of the drug. Intrathecal injection circumvents the BBB because the drug is delivered directly in the CNS [4–6]. Intrathecal injection has been used to deliver anesthetic agents for over a century [5, 7]. These injections are usually given at the lower lumbar levels to avoid spinal cord damage. However, the therapeutic targets are located throughout the CNS, including the spinal cord nerve roots and the supraspinal regions of the brain. The desired distribution of the drug depends on the therapeutic goals, which may range from localized delivery to broader dispersion along the spinal cord or even to supraspinal regions. A thorough understanding of the CSF dynamics and drug transport in the intrathecal space is essential to address the diverse targeting requirements in intrathecal delivery. Beyond drug delivery, investigating CSF motion is also important to mechanistically understand various CNS disorders that have been correlated with abnormal CSF motion [8–10]. A deeper mechanistic understanding of CSF dynamics within disease-specific environments, could enable better management and treatment strategies for CSF-related neurological disorders.

While the study of CSF dynamics has been dominated by invasive and non-invasive imaging techniques, computational modeling has emerged as a complementary tool that enables controlled manipulation of various parameters, such as, material properties and anatomical features. Earlier computational studies on CSF dynamics have considered CSF flow in multiple pathological situations [11–17] and have studied the impact of anatomical structures such as nerve roots [18, 19], trabeculae [20, 21] and denticulate ligaments [22] on the CSF flow. Recent computational models of the cranium have incorporated fluid–structure interaction (FSI) to capture the interactions between the parenchyma and SAS [23–26]. However, computational models of spinal CSF flow have assumed that the SAS is rigid, ignoring the key role of tissue compliance on CSF dynamics. Ignoring tissue compliance poses fundamental limitations in the predictive capabilities of the model, including the inability to accurately capture the timings of flow rate waveforms and the attenuation of CSF pulsations down the spine. This limitation is illustrated in [27], where fluid flow simulations were compared to 4D phase-contrast magnetic resonance images (PCMRI). Another fundamental limitation of fluid flow simulations of the spinal canal that ignore tissue compliance is that modeling pulsatile flow requires adding an artificial outlet boundary at the sacral end. This is a consequence of the incompressibility of CSF. Because CSF cannot be compressed or expanded, when we add or remove fluid from the spinal canal through a time-dependent boundary condition at the cranial end of the spine, the volume of the SAS needs to change accordingly. A common way to circumvent this problem is opening an outlet boundary in the spinal canal, but this is inaccurate and fundamentally changes the flow dynamics. More recent literature has attempted to model the effects of tissue compliance on CSF flow by imposing a pre-defined boundary motion on the walls of the SAS. Reference [28] has used this approach to simulate the effects of intrathoracic pressure changes caused by respiration. Similarly, in [29], the boundary motion was inferred from the phase lag and attenuation of the velocity peak amplitude of CSF velocity fields acquired from cine MRI. In reference [30], the authors used MRI-based nonuniform volumetric flow rates to prescribe radial displacements of the dura in 1 mm increments along the spinal canal. At each slice of the spinal canal, the difference between volumetric flow rate at the slice above and below determines the time-dependent radial displacement of the boundary. However, the approach based on pre-defined boundary conditions requires 4D MRI data and usually makes strong assumptions such as radially uniform displacement at each cross section of the spine. Reference [31] describes a computational model, where the dura mater is rigid, but the spinal cord is mobile and deformable. They found significant alterations in the flow with respect to flow simulations in a SAS that is time independent, but they also included an artificial outlet boundary in the caudal end of the spine, presumably because the potential compression and expansion of the spinal cord is not large enough to accommodate the fluid volume changes that occur during pulsatile flow in physiological conditions. Reference [32] used an anatomically idealized spinal SAS model with elastic dura mater showing that CSF steady streaming can be a major driving force of CSF bulk motion. However, in addition to the idealized geometry, the model is based on several approximations and uses a simplified linear elastic model for the outer wall of the SAS. To our knowledge fully coupled FSI simulations of the CSF and surrounding soft tissues have not been reported in the literature. One reason for the absence of such simulations is that accurate models of the mechanical response of the tissues surrounding the SAS requires specialized computational tools. The dura mater is a thin ($\sim \!200\,\upmu$m) membrane with relatively large Young’s modulus ($\sim \!4$ MPa) [33], while epidural tissue is thicker ($\sim \!4$ mm) and has a much smaller Young’s modulus ($\sim \!10^{-2}$ MPa). The combination of the two supporting tissues offers a complex mechanical response. Modeling dura mater as a volumetric solid requires very fine meshes, with at least 4-5 linear elements through the thickness, to avoid membrane locking. Even if the refinement is highly localized, it will inevitably propagate into the SAS and the epidural tissue, leading to a very large number of degrees of freedom.

Here, we propose a mixed-dimensional FSI model that addresses the aforementioned computational challenges and allows us to perform the first simulations of the entire spinal canal accounting for tissue compliance through a high-fidelity model of the soft tissues surrounding the SAS. In our mixed-dimensional approach, the dura mater is modeled as a curved membrane using surface kinematics, and epidural tissue is modeled as a volumetric solid. We perform simulations on a three-dimensional, anatomically accurate geometry of full-length human spine with closed caudal end. On the foramen magnum (FM), we impose a pulsatile velocity boundary condition obtained from MRI measurements. The flow from the pulsatile inlet boundary condition is accommodated by the deformations of the dura mater and epidural tissues. Using our FSI simulations, we describe the flow patterns, CSF velocities, pressure variations and the tissue displacements comparing them to experimental measurements. We show that the FSI simulation captures flow patterns that are more realistic than those from rigid-wall simulations that ignore tissue compliance. Finally, we examine the influence of tissue displacements on the cyclic mean velocities. Our simulations indicate that modeling of FSI is essential to capture the CSF flow patterns that are observed in vivo.

## Biophysical model

### Anatomical model

**Fig. 1:**
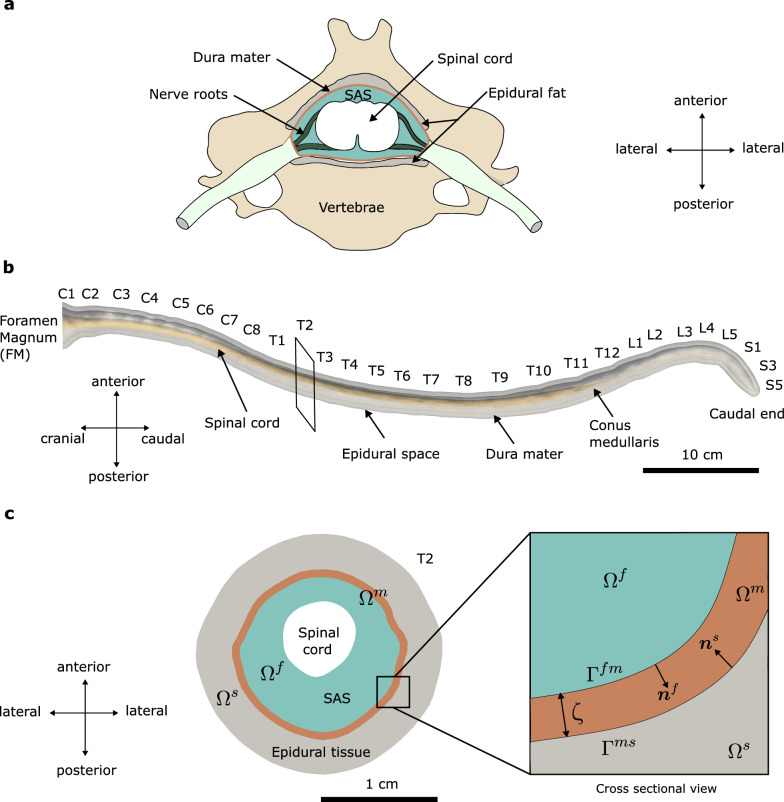
**a** Anatomical sketch of a cross-section of the spinal canal. **b** Geometrical model used in the current study. The geometry, obtained from [35], extends from the foramen magnum (FM) up to the closed caudal end. The spinal cord geometry (gold) is disregarded after the conus medullaris, where the cord narrows down to a very thin structure abruptly. **c** Cross-sectional view of the spine at the T2 level illustrating the different computational domains. The SAS (blue) is denoted by $\Omega ^f$, the dura mater (brown) by $\Omega ^m$, and the epidural tissue (gray) by $\Omega ^s$; note that the thickness of dura mater is not to scale. The interface between the CSF and dura mater is denoted by $\Gamma ^{fm}$, and the interface between dura mater and epidural tissue is denoted by $\Gamma ^{ms}$. The vectors $\varvec{n}^f$ and $\varvec{n}^s$ represent the unit outward normals to the boundaries of $\Omega ^f$ and $\Omega ^s$, respectively. The thickness of dura mater is spatially uniform and equals $\zeta = 0.5$ mm

Figure 1a illustrates a schematic cross-sectional view of the spinal canal. We model CSF flow within the SAS (blue) and its interactions with the surrounding solid tissues, including the dura mater (orange) and epidural fat (gray). In this study we disregard the presence of nerve roots (green), which may alter the flow patterns and provide structural support to the spinal cord (white). We also neglect CSF absorption within the SAS, which may occur around the nerve root sleeves because its influence is negligible relative to the dominant pulsatile component. We treat the spinal cord as rigid because the combination of its material properties and geometry make it much less deformable than dura mater or epidural fat. We assume that the epidural space solely contains fat, ignoring the presence of the venous plexus. The distribution of epidural fat along the spinal canal is uneven and discontinuous [34], but its precise geometry is not well characterized in the literature. Thus, in the absence of better data, we assume a uniform epidural tissue thickness of 4 mm throughout the spine.

We use triangulated surface-mesh files of the dura mater and spinal cord from [35] to construct the geometries of the SAS and the dura mater; see Fig. 1b. The geometries represent a full length anatomically accurate spine from the foramen magnum at the base of the skull to the coccyx. In Fig. 1b, we label the spine according to its vertebral levels, from C1 to S5, where C, T, L, S represent, respectively, the cervical, thoracic, lumbar, and sacral regions. In the sagittal plane, the spine displays significant curvature in three regions, whereas in the coronal plane, it appears nearly flat. The spinal cord geometry (gold colored in Fig. 1b) is included from foramen magnum down to the conus medullaris, where it tapers rapidly. We disregard the spinal cord geometry below the conus medullaris because its diameter is negligible and would not affect the CSF dynamics. We generate the geometry of the epidural tissue by uniformly extruding the dura mater surface outward by 4 mm along its surface normal; see Fig. 1b and the gray-shaded region in Fig. 1c.

### Computational domains and boundaries

The computational domain $\Omega$ consists of three subdomains such that $\Omega = \Omega ^f \cup \Omega ^m \cup \Omega ^s$. To avoid going into distracting mathematical details, we treat these subdomains as open or closed sets as convenient. Here, $\Omega ^f$ denotes the SAS, which is filled with CSF, $\Omega ^m$ represents the dura mater and $\Omega ^s$ is the epidural tissue. Unless otherwise specified all four domains evolve with time. The three subdomains $\Omega ^f$, $\Omega ^m$ and $\Omega ^s$ are spatially arranged as shown in Fig. 1b. Because the spinal cord is assumed to be rigid, it is not part of our computational domain. The SAS and the dura mater meet at a surface that we denote by $\Gamma ^{fm}$. The surface separating the dura mater and the epidural tissue is denoted by $\Gamma ^{ms}$. We call $\Gamma ^k$ the boundary of $\Omega ^k$ for $k=f,\, m,\, s$. The unit outward normal to $\Gamma ^k$ is $\varvec{n}^k$. Due to the topological arrangements of the subdomains it follows that $\varvec{n}^f = -\varvec{n}^m \text { on } \Gamma ^{fm}$ and $\varvec{n}^m = -\varvec{n}^s \text { on } \Gamma ^{ms}$.

### Cerebrospinal fluid flow

The strong form of the mass conservation and momentum balance equations in Eulerian description is

1 $$\begin{aligned} \rho ^f \dot{\varvec{v}}^f&- \nabla \cdot \varvec{\sigma }^f = \varvec{f}^f&\quad&\text {in } \Omega ^f\times [0,T], \end{aligned}$$

2 $$\begin{aligned} \nabla \cdot \varvec{v}^f&= 0&\quad&\text {in } \Omega ^f\times [0,T]. \end{aligned}$$

Here, $\rho ^f$ is the CSF density, $\varvec{v}^f(\varvec{x},t)$ is the velocity field, $\varvec{x}$ is a spatial point, *t* denotes time, [0, *T*] is our time interval of interest, ${\dot{\varvec{v}}}^f= \partial _t \varvec{v}^f + \varvec{v}^f \cdot \nabla \varvec{v}^f$ denotes the material derivative with respect to the fluid motion, and $\varvec{f}^f$ is a force per unit volume. We assume CSF can be modeled as a Newtonian fluid, thus

$$\begin{aligned} \varvec{\sigma }^f = -p^f\varvec{I} + \mu ^f (\nabla \varvec{v}^f + \nabla ^T \varvec{v}^f), \end{aligned}$$

where $\varvec{\sigma ^f}$ is the Cauchy stress tensor, $p^f(\varvec{x},t)$ is the pressure field, $\varvec{I}$ is the identity tensor $\mu ^f$ is the dynamic viscosity, and $\nabla ^T$ denotes the transpose operator of $\nabla$.

### Soft tissues

We model dura mater and epidural tissue as nonlinear hyperelastic materials with finite deformation kinematics. The elastodynamics equations in Lagrangian description read

3 $$\begin{aligned} \rho _0 \frac{\partial ^2 \varvec{u}}{\partial t^2} \bigg |_{\varvec{X}} - \nabla _{\varvec{X}} \cdot (\varvec{FS}) = \varvec{f}_0 \qquad \text {in } \Omega ^s_0 \cup \Omega ^m_0\times [0,T]. \end{aligned}$$

Here, $\rho _0$ is the solid mass density in the reference configuration, $\varvec{u}(\varvec{X},t)$ is the displacement field, $\varvec{X}$ denotes a material particle, $\varvec{F}= \nabla _{\varvec{X}} \varvec{u} + \varvec{I}$ is the deformation gradient, $\varvec{S}$ is the second Piola-Kirchhoff stress tensor, and $\varvec{f}_0$ is a force per unit volume of the reference configuration. The domains $\Omega ^s_0$ and $\Omega ^m_0$ represent, respectively, the reference configurations of $\Omega ^s$ and $\Omega ^m$. In Eq. (3), $\vert _{\varvec{X}}$ indicates that the time derivative is taken holding $\varvec{X}$ constant, and $\nabla _{\varvec{X}}$ indicates that the gradient operator is with respect to material particles. Because we will use different kinematics and different material models for dura mater and epidural tissue, we will use the displacement representation $\varvec{u}=\varvec{u}^s$ in $\Omega ^s_0$, and $\varvec{u}=\varvec{u}^m$ in $\Omega ^m_0$, where $\varvec{u}^m=\varvec{u}^s$ on $\Gamma ^{ms}$.

#### Kinematics and material model of the epidural tissue

We treat epidural tissue as a volumetric solid modeled as a compressible Neo-Hookean material. We adopt the strain energy density $\psi ^s$ with dilatational penalty from [36], given by,

4 $$\begin{aligned} \psi ^s = \frac{\mu ^s}{2} \left( J^{-2/3} \text {tr}(\varvec{C}) - 3 \right) + \frac{\kappa ^s}{2} \left( \frac{1}{2} (J^2 -1) -\ln J \right) . \end{aligned}$$

In Eq. (4), $\varvec{C}=\varvec{F^TF}$ is the right Cauchy Green strain tensor, $J = \text {det}(\varvec{F})$, $\operatorname {tr}(\cdot )$ denotes the trace operator, while $\mu ^s$ and $\kappa ^s$ are, respectively, the shear and bulk modulus of the tissue. The shear and bulk moduli can be obtained from the Young’s modulus ($E^s$) and the Poisson ratio ($\nu ^s$) as $\mu ^s = \frac{E^s}{2(1+\nu ^s)},\, \kappa ^s = \frac{E^s}{3(1-2\nu ^s)}$. For hyperelastic constitutive models, the second Piola-Kirchhoff stress tensor is obtained from the strain energy function as

5 $$\begin{aligned} & \varvec{S}^s = 2\frac{\partial \psi ^s}{\partial \varvec{C}} = \mu ^s J^{-2/3} \left( \varvec{I} - \frac{1}{3} \varvec{C}^{-1} \text {tr}(\varvec{C})\right) \nonumber \\ & \quad + \frac{1}{2} \kappa ^s \left( J^2 - 1 \right) \varvec{C}^{-1}. \end{aligned}$$

#### Kinematics and material model of dura mater

Unlike epidural tissue, the dura mater is very thin, and modeling it as a volumetric solid poses two challenges. First, we need sufficient mesh resolution through the dura thickness to avoid locking issues, which leads to an excessive proliferation of degrees of freedom. Second, even if meshing challenges are overcome the resulting mesh is highly non-uniform leading to linear systems with exceedingly large condition numbers. We avoid these challenges by using surface kinematics to describe the dura mater. We first express $\Omega ^m$ as

6 $$\begin{aligned} \Omega ^m = \Gamma ^m \times [0,\zeta ], \end{aligned}$$

where $\zeta$ is the thickness of the dura mater, and $\Gamma ^m$ is the mid-plane surface of dura. Because we assume that $\zeta$ is small, in our computations, we will identify $\Gamma ^m$ with $\Gamma ^{ms}$ or $\Gamma ^{fm}$ as needed to obtain solid displacements or impose the fluid-solid interface conditions.

Because the bending stiffness of dura mater scales with the cube of its thickness, we will neglect its resistance to bending. In our model, dura mater will behave as a membrane with stress restricted to in-surface components. Although such mechanical formulation would be unstable in its own, the restriction $\varvec{u}^m=\varvec{u}^s$ renders a stable formulation. We derive the in-surface stress tensor for dura mater following the approach for thin biological membranes described in [37].

**Fig. 2:**
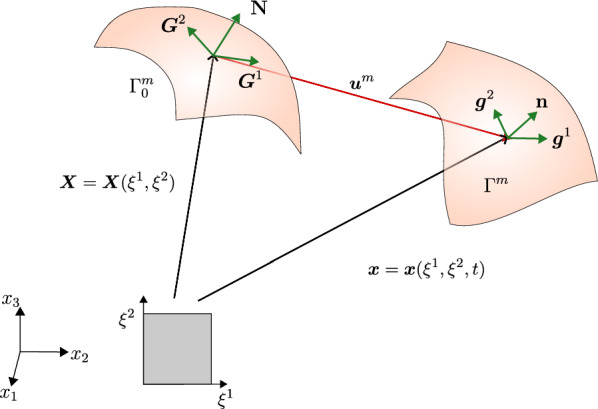
Surface kinematics for the dura mater membrane. The membrane in deformed and undeformed configurations is embedded in ${\mathbb {R}}^3$. The tangent vectors, $\varvec{G}^{\alpha }$ and the normal vector $\varvec{N}$ form the natural basis in undeformed configuration. The current configuration, obtained from the undeformed configuration by applying the displacement field $\varvec{u}^m$, has tangent vectors $\varvec{g}^{\alpha }$ and normal vector $\varvec{n}$

The reference configuration of dura mater is represented by the surface $\Gamma ^m_0$. We parametrize $\Gamma ^m_0$ with the mapping $\varvec{X} = \varvec{X}(\varvec{\xi })$, where $\varvec{\xi }=\{\xi ^\alpha \}_{\alpha =1,\,2}$ are curvilinear coordinates on the surface; see Fig. 2. The tangent vectors in the reference configuration are $\varvec{G}^\alpha = \partial \varvec{X}/\partial \xi ^\alpha$. The unit normal vector to $\Gamma ^m_0$ is obtained as $\varvec{N}=\varvec{G}^1\times \varvec{G}^2/||\varvec{G}^1\times \varvec{G}^2||$.

The deformed configuration of the dura mater is represented by the surface $\Gamma ^m$, which we parametrize with the mapping $\varvec{x} = \varvec{x}(\varvec{\xi }, t)$. Using the displacement field $\varvec{u}^m$, the tangent vectors for the deformed surface are $\varvec{g}^\alpha = \partial \varvec{x} / \partial \xi ^\alpha$; see Fig. 2.

To derive our constitutive model, we first decompose the total deformation gradient as

7 $$\begin{aligned} \varvec{F} = \varvec{F}_S + \varvec{F}_N \;\text { where }\; \varvec{F}_S = \varvec{g}_\alpha \otimes \varvec{G}^\alpha , \quad \varvec{F}_N = \lambda _N \varvec{n} \otimes \varvec{N}. \end{aligned}$$

In Eq. (7), $\lambda _N$ is the stretch in the direction normal to the undeformed surface and the repetition of Greek index $\alpha$ indicates summation. The surface deformation gradient $\varvec{F}_S$ is the surface projection of the deformation gradient $\varvec{F}$ and can be obtained using the projection tensor $\varvec{I}_S$ as

8 $$\begin{aligned} \varvec{F}_S = \varvec{F}\varvec{I}_S \text { where } \varvec{I}_S = \varvec{I} - \varvec{N} \otimes \varvec{N} = \varvec{G}_{\alpha } \otimes \varvec{G}^{\alpha }. \end{aligned}$$

Using Eq. (7), the right Cauchy Green strain tensor, $\varvec{C}$ can be split as

9 $$\begin{aligned} \varvec{C} = \varvec{C}_S + \varvec{C}_N \; \text { where } \; \varvec{C}_S = \varvec{F}_S^T \varvec{F}_S, \quad \varvec{C}_N = \lambda _N^2 \varvec{N} \otimes \varvec{N}. \end{aligned}$$

We model dura mater as an incompressible Neo-Hookean material with strain energy function

10 $$\begin{aligned} \psi ^m = \frac{\mu _m}{2}(\text {tr}(\varvec{C})-3) - \varsigma (J-1), \end{aligned}$$

where $\varsigma$ is the Lagrange multiplier for volumetric incompressibility. The second Piola-Kirchhoff stress tensor is

11 $$\begin{aligned} \varvec{S}^m = 2\frac{\partial {\psi ^m}}{\partial \varvec{C}} = \mu ^m \varvec{I} - \varsigma J \varvec{C}^{-1}. \end{aligned}$$

To obtain the value of the Lagrange multiplier, we impose the restriction that the normal component of $\varvec{S}^m$ be zero. The normal component of $\varvec{S}^m$ can be computed as $\varvec{S}^m_N = \varvec{S}^m \varvec{N}\otimes \varvec{N}$. From Eq. (9), we have $\varvec{N}\otimes \varvec{N}=\lambda _N^{-2}\varvec{C}_N$, thus, $\varvec{C}^{-1}\varvec{N}\otimes \varvec{N}= \lambda _N^{-2}\varvec{N}\otimes \varvec{N}$. Using this relation, we obtain

12 $$\begin{aligned} \varvec{S}^m_N = (\mu ^m - \varsigma J\lambda _N^{-2})\varvec{N}\otimes \varvec{N} \end{aligned}$$

Imposing $\varvec{S}^m_N=0$, we obtain $\varsigma =\lambda _N^{2}J^{-1}\mu ^m$. If we use $J=1=J_A\lambda _N$, where $J_A = ||\varvec{g}^1 \times \varvec{g}^2|| / ||\varvec{G}^1 \times \varvec{G}^2||$ is the surface stretch, we obtain the value of the Lagrange multiplier as $\varsigma =\mu ^m/J_A^2$.

After substituting the in-surface stress condition, we obtain the second Piola stress tensor with only surface contributions

13 $$\begin{aligned} \varvec{S}^m = \mu ^m (\varvec{I} - J_A^{-2} \varvec{C}^{-1}) =\varvec{S}^m_S. \end{aligned}$$

## Results

In this section, we present the flow predictions from our FSI simulation and compare them with those from a rigid-wall simulation and in vivo data from the literature.

### Problem definition and parameters

#### Prestress in tissue

We assume that the tissues that we model are in equilibrium with the traction from the fluid and other surrounding tissues at all times [38]. Thus, the geometry obtained from magnetic resonance imaging (MRI) is not stress-free. In the absence of better information, here, we assume a prestress $\varvec{\sigma }_0$ resulting from a mean pressure of $p_0=600$ Pa exerted by the CSF [39] and the outer surrounding tissues

14 $$\begin{aligned} \varvec{\sigma }_0 = -p_0\varvec{I} \text { at } t=0. \end{aligned}$$

#### Boundary conditions

**Fig. 3:**
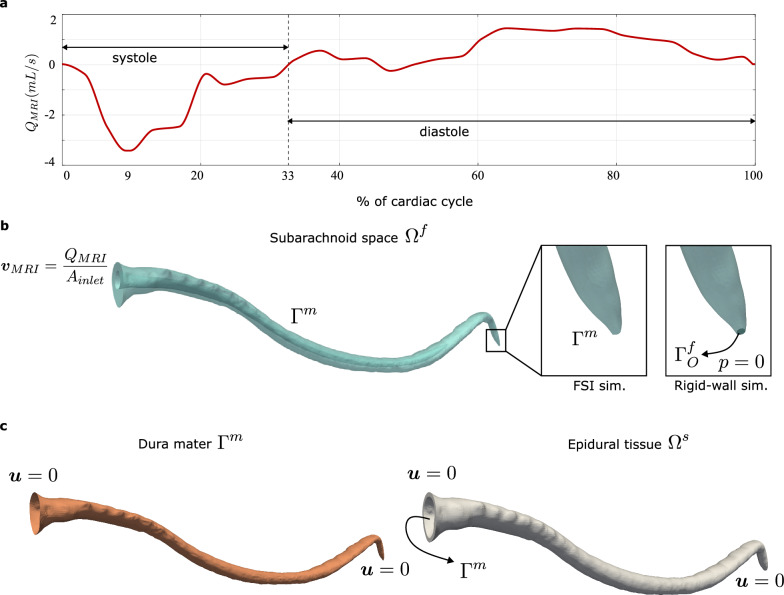
**a** Inflow waveform obtained from [35]. The waveform exhibits asymmetry between systole and diastole, with a dominant systolic peak occurring at approximately $t_\% = 9$ and a flow reversal at $t_\% = 33$. The stroke volume of the inflow waveform is 0.385 mL. **b** The inflow waveform from [35], is used to impose a velocity Dirichlet boundary condition $\varvec{v}_{MRI}$ on $\Gamma ^f_g$. In the FSI simulation, the CSF experiences traction from the lateral boundary $\Gamma ^m$. In the rigid-wall simulation, we use a slightly different geometry from the initial configuration of $\Omega ^f$. We open the caudal end to an outlet $\Gamma ^f_O$ and impose a zero-pressure condition. The inset figures compare how we treat the caudal end in the FSI and rigid-wall simulations. **c** We restrict the motion of tissues near the foramen magnum boundary and the caudal end. The dura mater experiences traction from either sides of the lateral boundary $\Gamma ^m$. The epidural tissue experiences traction from the dura mater and the outer surroundings

We obtain the CSF flow rate waveform at the C2 level ($Q_{MRI}$) from in vivo measurements reported in [35]. We assume a cardiac cycle period of $T_C=0.8$ s and adjust the waveform to ensure zero net volumetric flow over the cycle; see Fig. 3a. The stroke volume associated with the flow rate waveform (defined as the net CSF volume entering the spinal canal during systole) is 0.385 mL. Since the foramen magnum is located close to the C2 level, we assume that the flow rate waveform at the foramen magnum equals $Q_{MRI}$. Thus, we apply a time-dependent velocity Dirichlet boundary condition corresponding to the flow rate waveform $Q_{MRI}$ at the foramen magnum. We apply a flat, blunt-shaped velocity profile at the inlet boundary (Fig. 3b). We impose no-slip boundary conditions at the spinal cord wall.

We restrict the motion of tissues at the foramen magnum boundary in all directions (Fig. 3c). Additionally, a zero-displacement Dirichlet condition is applied at the caudal end to represent rigid support from the coccyx.

On the outer wall of the epidural tissue, we employ a traction boundary condition corresponding to the uniform pressure $p_0$. An alternative approach is to use the Robin boundary condition $\varvec{\sigma }^s \varvec{n}^s = -k\varvec{u} -c\varvec{v}-p_{0}\varvec{n}^s$ where *k* and *c* are parameters that represent the material behavior of the the tissues surrounding the epidural fat on the exterior side; see [40]. Because reliable experimental data for *k* and *c* are lacking, we varied *k* over a plausible range (with $c=0$) and found that the overall agreement with in vivo is best for $k=0$ (see Supplementary material). Hence, we adopt $k=0$ for the simulations reported in this work.

#### Initial conditions and material parameters

Our simulations were initiated with the conditions

15 $$\begin{aligned} \varvec{v}(\varvec{x},0)&= \varvec{0}; \quad \varvec{x} \in \Omega ^f, \end{aligned}$$

16 $$\begin{aligned} p(\varvec{x},0)&= 600 \text { Pa}; \quad \varvec{x} \in \Omega ^f, \end{aligned}$$

17 $$\begin{aligned} \varvec{u}(\varvec{x},0)&= \varvec{0}; \quad \varvec{x} \in \Omega ^s. \end{aligned}$$

Because the above initial conditions are unrealistic, we ran the simulations until the flow field became periodic in time. We found that the flow became periodic with 92.4% accuracy after 5 cardiac cycles. We report results for the $6^{\text {th}}$ cycle. To identify time in our results, we will use $t_\%\in [0,100]$, which represents the percentage of the cardiac cycle in the 6th cycle. Table 1 lists the material properties used in this study.

**Table 1:** Material properties used in our simulations

Parameter	Symbol	Value	Unit	References
Cerebrospinal fluid (CSF)				
Density	$\rho ^f$	1000	kg/$\hbox {m}^3$	[41]
Viscosity	$\mu ^f$	$10^{-3}$	Pa$\cdot$s	[42]
Dura mater				
Young’s modulus	$E^m$	4.0	MPa	[33, 43]
Poisson’s ratio	$\nu ^m$	0.5	–	–
Thickness	$\zeta$	0.2	mm	[33]
Epidural fat				
Young’s modulus	$E^s$	10	kPa	[44]
Poisson’s ratio	$\nu ^s$	0.4	–	–
Density	$\rho _0$	1000	kg/$\hbox {m}^3$	–
Thickness	–	4	mm	–

#### Rigid wall simulations

To better understand the effect of tissue compliance, we also performed a rigid-wall simulation for comparison purposes. In our rigid-wall simulations, all solid tissues are assumed to be undeformable and do not need to be included in the computational domain. The rigid-wall simulation was performed on the domain $\Omega ^f_{\text {rw}}$, which is slightly different from the initial configuration of $\Omega ^f$. The motivation to modify the fluid domain is that rigid fluid domain is incompatible with the pulsatile inlet velocity that we impose at the foramen magnum because the fluid is incompressible. The most common approach in the literature to avoid this incompatibility is to open an outlet in the sacral region of the domain; see $\Gamma _O^f$ in the inset in Fig. 3. We impose a zero pressure boundary condition on $\Gamma _O^f$, which allows the fluid to exit the domain to satisfy mass conservation.

### Flow patterns

To analyze the flow patterns along the spine, we define $d=-z$ as the distance from the foramen magnum. At a distance *d* from the foramen magnum, we define the cross sections of the SAS ($\Gamma ^f_d$) as the intersection of $\Omega ^f$ with the plane $z=-d$. The flow rate across the cross section defined by *d* is given by $Q_d (t) = \int _{\Gamma ^f_d}v_z(t) \text {d} a$.

**Fig. 4:**
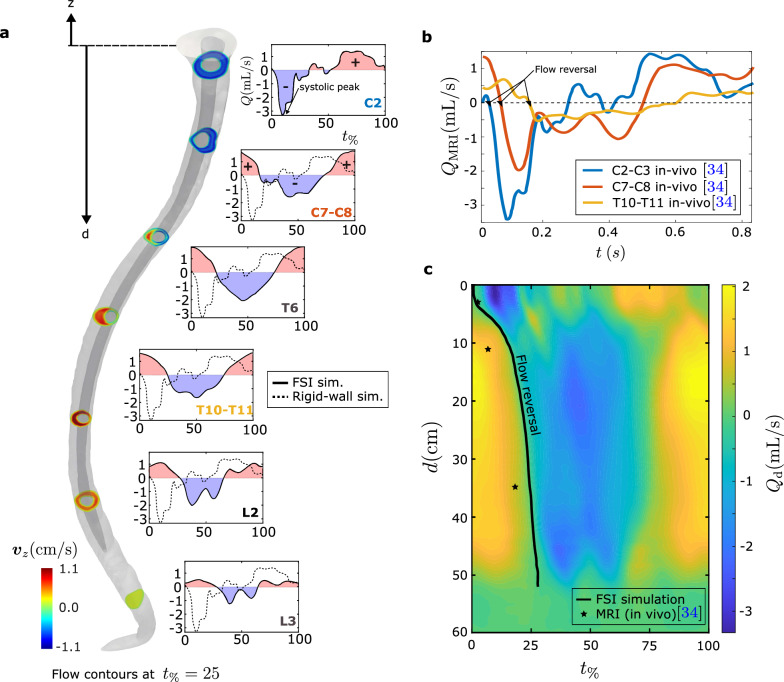
**a** Flow rate waveforms at various locations obtained from our FSI simulation (solid line) and rigid-wall simulation (dashed line). Blue shade represents flow in caudal direction (−), red shaded region represents flow in cranial direction ($+$). The contours in sections along spine indicate the *z*-component of velocity at $t_{\%}=25$. **b** In vivo measurements of flow rate waveforms from [35] (we also used [35] to obtain the geometrical model of the spine). **c** Spatio-temporal distribution of volume flow rates from FSI simulation over a cardiac cycle. The negative flow rate denotes the flow is craniocaudal, and positive flow rates denotes caudocranial flow. The stars mark the flow reversal timing based on the in vivo observations [35] at C2-C3, C7-C8 and T10-T11 levels. We show the flow reversal in our simulation through the black curve $(Q_d=0)$, which also occurs at different times for different locations

In the rigid-wall simulation, the flow rate waveforms across all sections are identical to the inflow waveform. However, in the FSI simulations and in the in vivo measurements they vary as we move along the spine because the fluid domain is deforming with time. Figure 4a shows the flow rate waveforms at various sections. The red shaded region represents positive flow rates, indicating cranial flow, while the blue shaded region corresponds to negative flow rates, indicating caudal flow. The waveforms obtained from our FSI simulations vary noticeably across the sections, consistent with in vivo measurements from MRI taken at levels C2-C3, C7-C8, and T10-T11 [35]; see Fig. 4b.

The flow rate waveforms predicted in the FSI simulation capture two key features that are consistent with in vivo data: phase differences and craniocaudal decay in pulsation amplitude. These two aspects are analyzed separately in what follows.

#### Flow rate waveform phase differences

The phase differences in the FSI simulation can be quantified by tracking two key characteristics of the flow rate waveforms along the spine. The first one is the systolic peak: the time when the flow rate at a section reaches its maximum during a cycle. The second one is the flow reversal time: The time at which the flow direction reverses at the section. By recording the systolic peak along the spine, we can calculate the pulse wave speed (PWS), which is defined as the ratio of the distance traveled by the systolic peak to the time it takes to travel that distance. The PWS measures the compliance of the tissue and has been reported in multiple experimental papers based on MRI data. The PWS in our FSI simulation is spatially non-uniform, with an average value of 3.4 m/s, which is within the range of measured values in the literature; see Table 2. At the section defined by $z=-d$, the phase difference, $\Delta \phi (d)$, between the flow rate waveform $Q_d$ at that section and the inflow waveform ($Q_{MRI}$) can be estimated as $\Delta \phi (d) = 2\pi \frac{d}{\text {PWS} \times T_C}$, where $T_C$ is the time period of the cardiac cycle.

**Table 2:** Comparison of the pulse wave speed (PWS) obtained from our FSI simulations and the PWS obtained from in vivo measurements

Study	PWS (m/s)	Type of study
Current	3.4	Computational
Sass et al. (2017) [35]	1.94	In vivo
Khani et al. (2017) [30]	1.19	In vivo
Sonnabend et al. (2021) [45]	5.83±3.36	In vivo
Kalata et al. (2009) [46]	4.6±1.7	In vivo

Simulations that ignore tissue compliance predict an infinite PWS

The phase difference in the flow rate waveform at an intermediate location of the spine is also related to the time delay in flow reversal ($Q_d=0$) relative to the foramen magnum. Figure 4b depicts in vivo flow rate data from [35] showing that the flow reversal time changes from one section to another. Using the data from our FSI simulation, we produced a spatio-temporal distribution of the flow rates from the FSI simulation; see Fig. 4c. The star markers in Fig. 4c represent in vivo flow reversal times, while the black solid line defines the space-time curve of flow reversal predicted by our simulation. Our FSI simulation captures the spatial variation in flow reversal timing more accurately than the rigid-wall simulation. In the rigid-wall simulation, flow reversal occurs simultaneously at all locations, and the data would define a vertical straight line in Fig. 4c (not shown). Note that the flow rate waveforms in the lumbo-sacral regions are distorted with respect to the inflow waveform due to the pulse wave reflections near the caudal end. Hence, the descriptions of systolic peak and flow reversal in these regions are not meaningful.

#### Craniocaudal decay of the flow rate

Due to the tissue compliance, the pulse wave energy associated with the inflow waveform attenuates as it travels along the spine. To quantify the craniocaudal decay in CSF pulsations, we calculate the peak flow rate, which represents the amplitude of the flow rate waveform at a cross section.

**Fig. 5:**
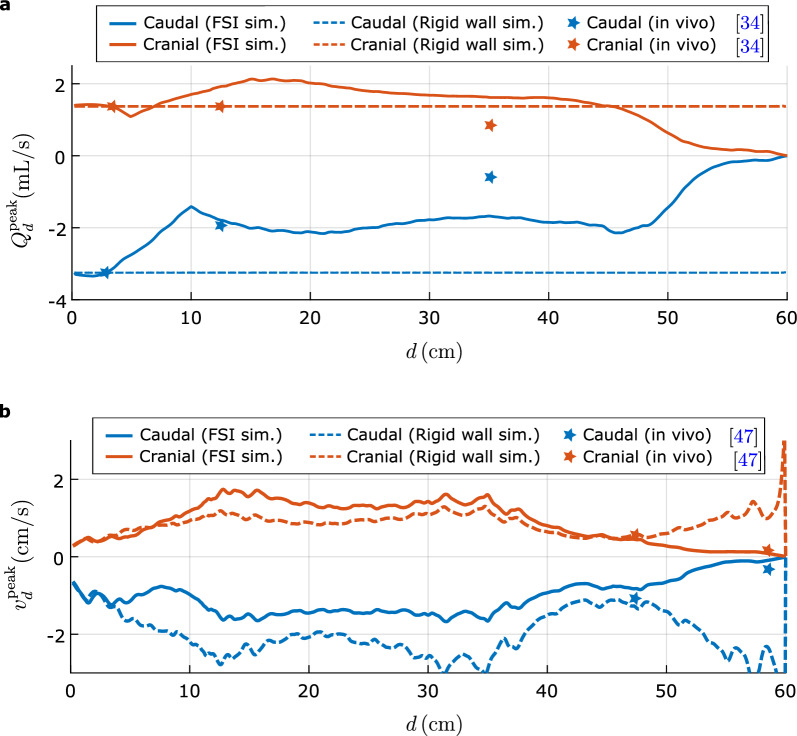
**a** Caudal (red) and cranial (blue) peak flow rates from FSI simulations (solid lines), rigid-wall simulations (dashed lines) and in vivo measurements (stars). The in vivo data are taken from [35]. **b** Peak velocity, $\varvec{v}^{\text {peak}}_d$ from the FSI simulation, the rigid-wall simulation and from in vivo measurements. The in vivo data are taken from [47]

The peak flow rate in the cranial and caudal direction is defined as $Q_{d,\text {cra}}^{\text {peak}}=\max _{t_\%\in [0,100]} Q_d(t)$ and $Q_{d,\text {cau}}^{\text {peak}}=\min _{t_\%\in [0,100]} Q_d(t)$ respectively. Figure 5a compares the peak flow rates obtained from our FSI simulation (solid lines), with those obtained from our rigid-wall simulation (dashed lines) and with the MRI observations (stars). In the rigid-wall simulation, $Q_{d}^{\text {peak}}$ remains constant as *d* varies and always matches the flow rate imposed by the inlet boundary condition. However, in the results from the FSI simulation and the in vivo measurements, the peak flow rates varies along the spine. In the FSI results, the asymmetry between the peak cranial and peak caudal flow rate associated with the inflow waveform is small at the intermediate sections. For example, at $d=10$ cm, the peak caudal and cranial volume flow rates are nearly equal ($\approx 1.8$ mL/s), and match the values reported in vivo (1.95 ml/s); see Fig. 5a. Overall, this plot illustrates the inability of the rigid-wall model to capture the craniocaudal decay of the flow rate.

To further study the CSF flow velocities, we compare the peak velocities from our FSI simulations with those from rigid wall simulations and with in vivo experiments. We define the caudal and cranial peak velocity at distance *d* from the foramen magnum as

18 $$\begin{aligned} & v^{\text {peak}}_{d,\text {cau}} =\max _{t_\%\in [0,100]} \frac{ \int _{\Gamma _d^f} \varvec{v}\cdot \varvec{n}_d \text {d}a }{ \int _{\Gamma _d^f} \text {d}a }, \nonumber \\ & \quad v^{\text {peak}}_{d,\text {cra}} =\min _{t_\%\in [0,100]} \frac{ \int _{\Gamma _d^f} \varvec{v}\cdot \varvec{n}_d \text {d}a }{ \int _{\Gamma _d^f} \text {d}a }, \end{aligned}$$

where $\varvec{n}_d$ is the normal to $\Gamma _d^f$ in the cranial direction. Figure 5b shows $v^{\text {peak}}_d$ from our simulations. Because the spine is closed at the caudal end in the FSI simulation, the peak velocities in the lower lumbar and sacral regions predicted by the FSI simulation are significantly smaller than those from the rigid-wall simulation. In the lumbar region, the value of ${v}_{d,\text {cau}}^{\text {peak}}$ obtained from the FSI simulation ranges from 2 cm/s to 0.25 cm/s, while in the sacral region is less than 0.25 cm/s. These results agree well with the experimental literature [47] (see stars in Fig 5b), where peak velocities of $-1.07\pm$0.49 cm/s and $-0.32\pm$0.33 cm/s are reported at the L2 and S1 levels, respectively.

### Intraspinal pressure

We analyze the time variation of the average pressure at cross sections of the spine. We call this quantity the intraspinal pressure (ISP), which at the cross section defined by $z=-d$, is computed as

19 $$\begin{aligned} \text {ISP}_d(t) = \frac{\int _{\Gamma ^f_d}p(t) \text {d} a}{\int _{\Gamma ^f_d} \text {d} a}. \end{aligned}$$

**Fig. 6:**
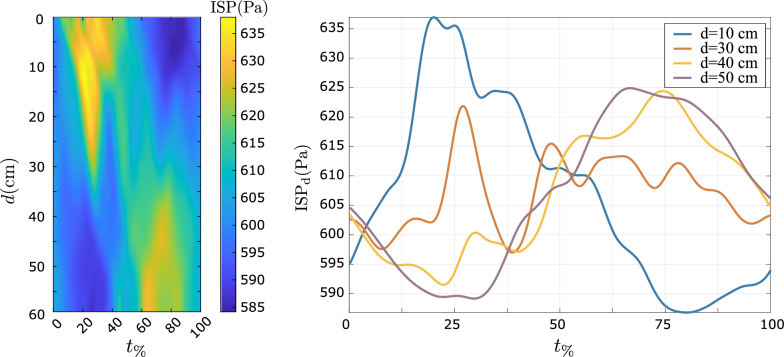
Left: Spatio-temporal distribution of absolute pressure. Note the pressure wave reflections occurring at the sacral end at $t_\%\approx 40$ and $t_\%\approx 60$. Right: Mean pressure waveforms at different cross-sections of the spinal canal. The pressure waveforms at the lower spine levels are distorted due to wave interactions with the reflected wave from caudal end

In the FSI simulation, the absolute pressure depends on the pressure initial condition and the prestress in the tissue. Hence, we only analyze the temporal and spatial variations of the pressure field. Figure 6 shows how the $\hbox {ISP}_d$ varies with *d* and time in our FSI simulation. During the systolic phase, the pressure is higher in the cervical regions, and decreases toward the caudal end. At $t_\%=50$, the pressure field becomes nearly uniform in space. In the latter half of the cycle, the pressure in the cervical regions begins to drop, resulting in pressure gradient in the cranial direction. By the early systole of the next cycle ($t_\%\approx 5$) the pressure gradient reverses, with the pressure higher in the cervical regions relative to the lower spinal levels.

In the FSI simulation, at any time in the cardiac cycle, the pressure difference between the foramen magnum and the caudal end does not exceed 60 Pa. By comparison, the maximum pressure difference is 140 Pa in the rigid-wall simulation. For validation, we refer to a study where the pressure was measured invasively using a catheter at the lumbar region. The amplitude of pressure at L2 level in the FSI simulation is 35 Pa (Fig. 6), which agrees with the clinical range of 4-10 mm $\hbox {H}_2$O ($\approx$ 39-98 Pa) [48].

The pressure waveform at different levels exhibits a phase lag relative to the pressure waveform at the foramen magnum. This phase lag increases caudally, reaching approximately $\pi$ at the caudal end. However, in the rigid-wall simulations, the pressure waveforms at all locations are in-phase.

In contrast to the rigid-wall simulations, where the pressure waves cannot be captured due to infinite speed, in the FSI simulations, we observe several wave reflections near the caudal end (Fig. 6). Successive reflections occur at $t_\% \approx 40$, and $t_\% \approx 60$. However, such wave reflections cannot be captured through MRI because they produce only small flow deviations, which are below the detection threshold.

### Tissue displacements

The pulsatile motion of CSF in the spinal canal causes deformations in dura and epidural fat. Here, we are interested in two quantities: (i) percentage change in cross sectional area of SAS, and (ii) maximum tissue displacements. We calculate the maximum percentage change in area of cross-section of SAS due to the tissue deformations over the cardiac cycle as

20 $$\begin{aligned} {\% \Delta A^{SAS}_d = \frac{\max \limits _{t_\%\in [0,100]} \bigg (\int _{\Gamma ^f_d(t_\%)} \text {d} a \bigg ) - \int _{\Gamma ^f_d(t=0)} \text {d} a}{\int _{\Gamma ^f_d(t=0)} \text {d} a} \times 100.} \end{aligned}$$

**Fig. 7:**
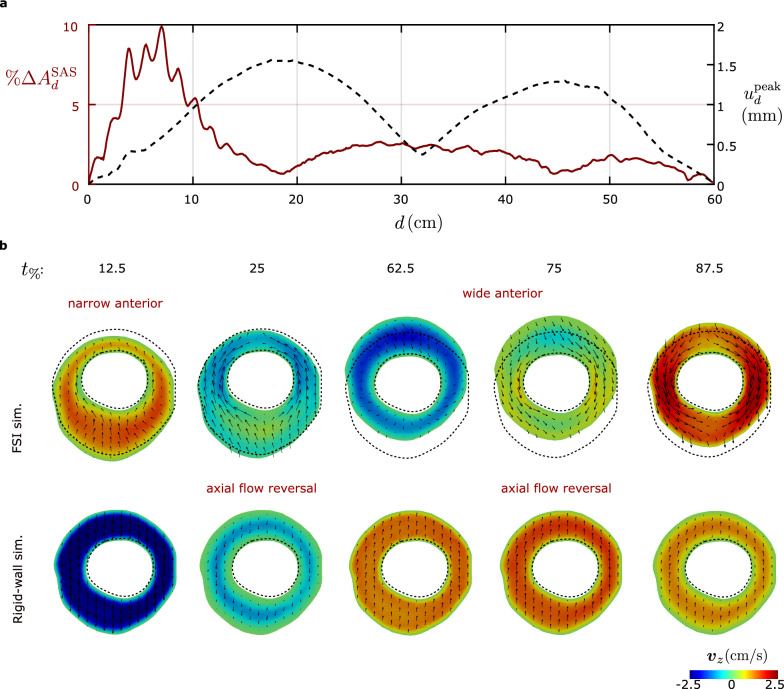
**a** Area deformations and tissue displacements at different distances from foramen magnum. **b** Tissue deformations altering the fluid flow significantly. The azimuthal flow velocities, represented by the sizes of the glyphs are larger in FSI simulations compared to those in rigid-wall simulations. Note the tissue deformations nearly reverses the geometrical features of SAS anterior-posterior from $t_\%=12.5$ to $t_\%=62.5$

The maximum tissue displacement is calculated as $u^{\text {peak}}_d\! =\max _{\varvec{x}\in \Gamma _d^f} \max _{t_\%\in [0,100]} {\Vert \varvec{u}\Vert }$. Figure 7a plots $\%\Delta A_d^{SAS}$ and $u_d^{\text {peak}}$ with respect to distance from the foramen magnum. The maximum $\%\Delta A^{SAS}_d$ occurs at C5 level and takes the value $10\%$, which corresponds to an absolute change of $\sim$22 $\text {mm}^2$ in the cross section. Secondary maxima occur near the T9 and L3 levels. We observe large tissue displacements, with a maximum of 1.5 mm occurring at the T4 level, i.e., $d\approx$ 19 cm; see Fig. 7a. At the T4 level, however, $\%\Delta A^{SAS}_d$ is very small. In general, the large tissue displacements occur predominantly with rigid body motions of the cross sections, and do not contribute significantly to $\%\Delta A^{SAS}_d$.

Figure 7b, highlights the cross section where the rigid body displacements in the tissue are the largest ($d\approx 19$ cm). The dashed lines indicate the boundary of dura mater in the undeformed configuration. We display the *z*-component of the velocity using a colormap to indicate axial flow direction. In the anterior side of spine, the SAS narrows during the systole ($t_\%=12.5$), and widens during diastole ($t_\%=62.5$). Conversely, in the posterior spine, the SAS widens during systole and narrows by mid-diastole.

The black arrows in the Fig. 7b illustrate the in-plane velocity vectors. The in-plane velocities in the FSI simulation are larger than those in the rigid-wall simulation due to the motion of the dura mater. For instance, at $t_\%=12.5$, the dura displaces in the anterior direction, therefore pushing the CSF in the anterior direction. Whereas, at $t_\%=75$, the dura displaces towards the posterior direction, pushing the CSF from anterior to posterior side.

To our knowledge, there is no experimental literature on dura deformations, hence we could not compare our model results in terms of tissue deformations. However, since we did not account for the mechanical support for the epidural fat, the large displacements observed (up to 1.5 mm) may be an overestimate. In actual physiology, the distribution of epidural fat is highly discontinuous and is backed by the rigid bone. Modeling tissue support around the epidural fat would require precise knowledge of the support locations.

### Steady-streaming

Pulsatile flow in a deformable channel results in a non-zero mean flow over a cycle. This phenomenon is referred to as steady-streaming [49, 50]. In the radionuclide scanning studies [51, 52], the authors observed that a tracer injected in the lumbar region moves upward and reaches the basal cisterns in 15-20 min. This observation indicates net upward motion of the tracer over one cardiac cycle with velocities in the order of 1 cm/min, which is significantly faster than the velocities expected from diffusion. The precise reason for this rapid net upward motion of tracers is multifaceted and poorly understood. Recent studies [32, 53] on steady-streaming in simplified geometrical models of the spine have shown that, under certain assumptions, the cumulative effects of nonlinear convective accelerations result in a CSF bulk motion consistent with the observations from radionuclide scanning studies [51, 52]. Here, we explore the steady-streaming in our simulations due to the nonlinear convective acceleration and tissue deformation in an accurate three-dimensional patient specific geometry.

Steady-streaming has been quantified in the literature using the Eulerian and Lagrangian descriptions. The Eulerian steady-streaming velocity is the cyclic time averaged velocity at fixed points in space. When the fluid domain moves with time, as in our FSI simulation, the exact definition of Eulerian steady-streaming velocity becomes inapplicable because some regions outside of the initial configuration can have active mean velocities. The concept of Eulerian steady-streaming velocity can be extended to moving domains in multiple ways, none of them completely satisfactory. Here, we define the Eulerian steady-streaming velocity as the cyclic time averaged velocity as observed from points fixed with respect to the moving domain,

21 $$\begin{aligned} \langle \varvec{v} ({\widehat{\varvec{x}}}) \rangle _{E} = \frac{1}{T_c}\int _0^{T_c} \varvec{v}(\widehat{\varvec{x}},t) \,\text {d}t. \end{aligned}$$

The Lagrangian steady-streaming velocity is the time averaged velocity of a fluid particle along its trajectory over a cardiac cycle,

22 $$\begin{aligned} \langle \varvec{v}(\varvec{X}) \rangle _{L} = \frac{1}{T_c}\left[ \varvec{X}(t+T_c) - \varvec{X}(t)\right] . \end{aligned}$$

We carried out the calculations in Eqs. (21) and (22) using PyVista [54]. Both the Eulerian and Lagrangian steady-streaming velocity fields in our FSI simulation are nearly identical (not shown here), therefore we only report the steady-streaming fields in Eulerian description.

**Fig. 8:**
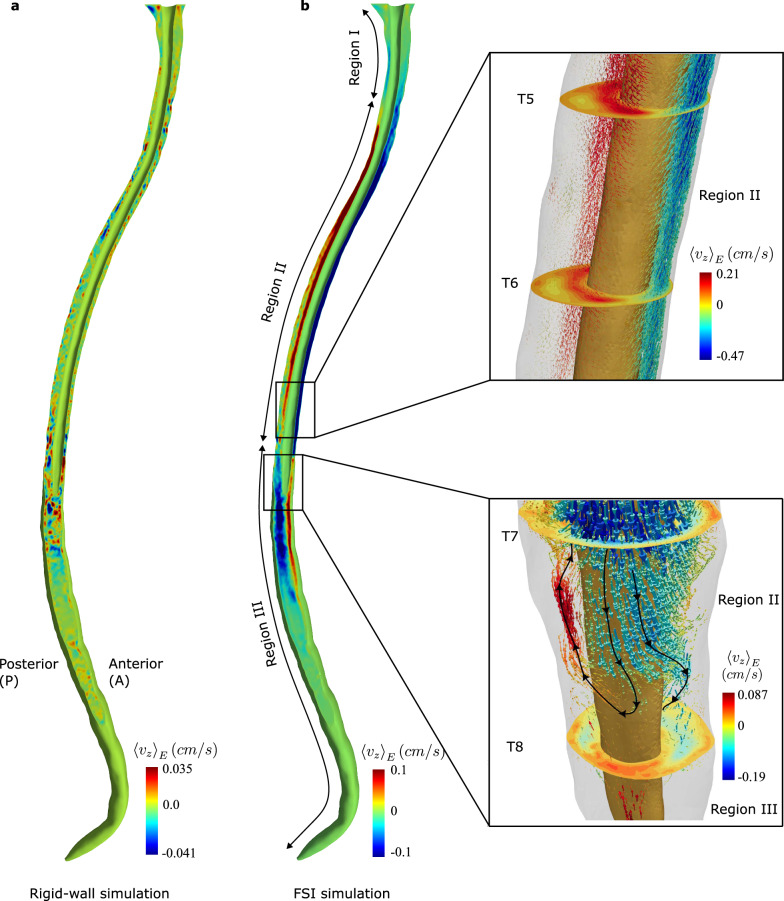
**a** Sagittal view of the steady-streaming velocities obtained from the rigid-wall simulation. We could not identify any recognizable pattern in the rigid-wall steady-streaming velocities. **b** Sagittal view of steady-streaming velocities from the FSI simulation. The FSI simulation shows larger steady-streaming velocities than the rigid-wall simulation. The regions I, II, III are defined based on the steady-streaming directions of the FSI data. The steady-streaming is very small close to the foramen magnum due to the boundary condition, and almost negligible in the lumbosacral regions due to small pulsatile velocities. Top inset: The steady-streaming velocity glyphs in Region II show bi-directional flow with azimuthal flow on the lateral side of spine. Bottom inset: The steady-streaming velocity glyphs near the separation line show velocity glyphs returning into Region II as they approach Region III

Figures 8a and 8b show the *z*-component of steady-streaming velocities from our rigid-wall simulation and our FSI simulation, respectively. Negative and positive velocity represent, respectively, caudal and cranial flow. The steady-streaming velocities in the rigid-wall simulation are smaller than those in the FSI simulation and do not exhibit any sensible pattern. In the FSI simulation, steady-streaming velocities show a bi-directional flow, with upward (cranial) steady-streaming on one side (anterior or posterior) and downward steady-streaming on the other side. These flow directions alternate along the length of the spine; see Fig. 8b. We name the regions with alternating patterns as I, II and III; see Fig. 8b. The inset figures (top and bottom) in Fig. 8b show the 3D glyphs of the steady-streaming velocity inside II, and near the intersection between II, III. The glyph size is scaled to the *z*-component of the steady-streaming velocity. The flow in II is bi-directional with upward steady-streaming in the posterior side, and downward in the anterior side. In the lateral side of spine in II, we observe that the flow is predominantly azimuthal, with velocities of nearly the same order of magnitude as the axial steady-streaming velocities.

The flow patterns in II agree with those reported in [32, 53], where the alternating bi-directional flow regions are identified as vortices divided by separation lines. In our simulation, we do not observe clear vortex type behavior; see bottom inset in Fig. 8b. The black line with arrows illustrates the flow direction near the intersection between II and III. The caudal flow in anterior side of II deviates azimuthally as it approaches III and only a part of the flow returns back into the II, while rest of the flow passes into III. Moreover in [32, 53], the authors show that the eccentricity of the spinal cord made with dura mater is a key geometrical feature that determines the location of separation lines. However in our simulation the eccentricity of spinal cord changes significantly with time, and we observe the separation lines near C5-C6 and T8-T9 levels, which are located slightly above the levels where the eccentricity of spinal cord in initial configuration reverses.

To put the magnitudes of steady-streaming velocity in context, in our FSI simulation the spatial maximum of $\langle \varvec{v} \rangle _{E}$ is 0.54 cm/s in the caudal direction, which occurs near the T5 level. Because the pulsatile velocities in the FSI simulation are smaller at the lumbar and sacral levels, we observe smaller steady-streaming velocities (order of 0.001 cm/s) relative to those in the cervical and thoracic levels. To our knowledge, there is no experimental study that has characterized steady-streaming velocities due to their small value, hence we could not be compare them with experiments. Comparing our steady-streaming velocities with the radionuclide scanning observations at this stage is premature because tracer transport needs to account for several factors such as binding and degradation.

To further quantify the effects of tissue deformation on steady-streaming, we evaluate the steady-streaming flow rate across a section in the upward or downward direction. The steady-streaming flow rates in the upward direction at the section $z=-d$ are defined, respectively, as

23 $$\begin{aligned} Q^{\text {SS}}_d = \int _{\Gamma _d^f} \max (\langle v_z \rangle _{E},0) \text {d}a. \end{aligned}$$

**Fig. 9:**
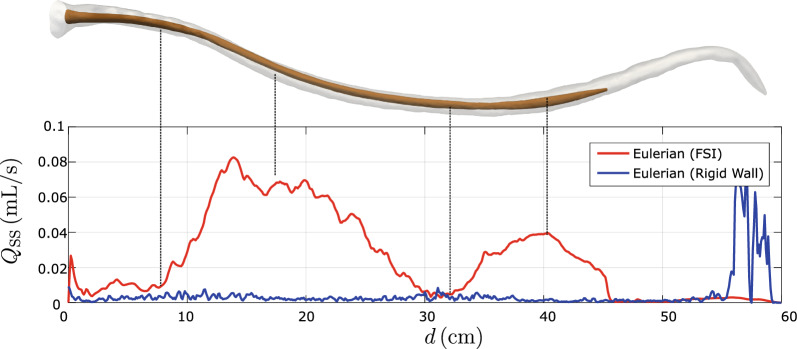
Comparison of steady-streaming strength from the FSI simulation (red line) and the rigid-wall simulation (blue line). In the FSI data, regions with maximum steady-streaming correspond to locations of large tissue deformations, while a minimum occurs near the T8 level, where the separation line is located. In the rigid-wall simulation, there is no recognizable pattern, except large oscillations at the sacral end, which are due to the (unavoidable) outlet boundary condition

Figure 9 compares $Q^{\text {SS}}_d$ in rigid-wall simulations with those in FSI simulations. The $Q^{\text {SS}}_d$ in FSI simulation is $\sim$10 times larger than in the rigid-wall simulation. In the FSI data, $Q^{\text {SS}}_d$ exhibits local maxima at $d\approx 14$ cm and $d\approx 40$ cm, where the tissue displacements are largest; and local minima at $d\approx 31$ cm, where the steady-streaming flow direction is non-axial.

Understanding the Eulerian and Lagrangian steady streaming velocities of CSF can reveal key factors influencing drug transport. For instance, identifying regions with minimal steady streaming such as separation lines can aid in optimizing drug delivery procedures. Additionally, the Eulerian cyclic mean velocities are used as frozen velocity field in [28] when simulating drug transport that requires computations of oscillatory flow fields across thousands of cycles.

## Conclusion

We proposed a mixed-dimensional computational FSI model to simulate the CSF flow dynamics and tissue mechanics in a 3D patient specific model of the spinal canal. Due to the different length scales of dura mater and epidural fat, we discretized dura mater as a 2D membrane, and epidural fat as a 3D solid. To emphasize the critical role of tissue compliance in the CSF dynamics, we compared our FSI simulations with rigid-wall fluid flow simulations. Our FSI simulation revealed several key flow mechanisms that the rigid-wall simulations failed to capture: (1) Phase lag between the flow rates at different sections of the spine, corresponding to a finite pulse wave speed of 3.4 m/s that agrees with in vivo measurements, (2) Craniocaudal decay in flow pulsations with small velocities in the caudal region, (3) Strong and well-defined bi-directional flow pattern of steady-streaming velocities qualitatively compatible with previous experimental [51, 52] and theoretical [32, 53] studies.

Our model could be further developed in several ways. First, incorporating the nerve roots, which alter the details of the CSF flow dynamics. Second, accounting for the compliance of the spinal cord. Considering the deformation of the spinal cord and its mechanical connection to the dura mater through the nerve roots would likely reduce pulse wave speed, and increase the phase lags in flow rate and pressure waveforms. Third, using a more detailed anatomical and physiological description of the epidural fat, including its interactions with surrounding tissues and the presence of the venous and arterial plexus. The arterial and venous plexus affect the CSF motion by transmitting the intra-thoracic or intra-abdominal pressure fluctuations. The effects of arterial/venous plexus could be modeled by using a time-dependent traction boundary condition on the outer wall of the SAS, with experimental pressure data.

We anticipate that our computational method will open new possibilities to better understand structural abnormalities of the CNS. Perhaps more importantly, it can enable control and optimization of intrathecal drug delivery techniques which will lead to more effective therapies for CNS disorders.

## Supplementary Information

Supplementary material 1.

## Data Availability

The data supporting the findings of this study are available within the paper.

## Appendix

### Computational method

We adopt a boundary-fitted approach for our FSI formulation. The governing equations of fluid and solid mechanics are discretized in their corresponding domains using the finite element method. The discretization of the fluid and solid domains match at the fluid-solid interface $\Gamma ^{m}$. Time discretization is performed using backward Euler. The fluid problem occurs on a time-evolving domain. The Eulerian form of the fluid dynamics equations presented in Sect. 2.3 is not well suited for semi-discretized methods on moving domains. Because we are interested in using a semi-discrete approach we will rewrite the fluid dynamics equations in Arbitrary Lagrangian–Eulerian (ALE) formulation.

#### Arbitrary Lagrangian–Eulerian (ALE) formulation of the fluid problem

In the ALE approach, a static referential domain is introduced, defined by the reference coordinates. The spatial domain at time *t* is obtained by deforming the referential domain through a mapping described by the mesh displacement $\widehat{\varvec{u}}(\cdot ,t)$. Using the techniques presented in [55, 56], we write the strong form of CSF flow problem in ALE description as,

24 $$\begin{aligned}&\rho ^f \frac{\partial \varvec{v}^f}{\partial t} \bigg |_{\widehat{\varvec{x}}} + \rho ^f(\varvec{v}^f-\widehat{\varvec{v}})\cdot \nabla \varvec{v}^f - \nabla \cdot \varvec{\sigma }^f = \varvec{f}^f \qquad&\text {in } \Omega ^f\times [0,T],\end{aligned}$$

25 $$\begin{aligned}&\nabla \cdot \varvec{v}^f = 0 \qquad&\text {in } \Omega ^f\times [0,T]. \end{aligned}$$

where $\varvec{{\widehat{v}}}=\partial _t{\widehat{\varvec{u}}}$ is the velocity of fluid mesh motion and the subscript $\widehat{\varvec{x}}$ in $\frac{\partial \varvec{v}^f}{\partial t} \big |_{\widehat{\varvec{x}}}$ indicates that the time derivative is taken by holding the reference coordinates (${\widehat{\varvec{x}}}$) fixed. Note, however, that all spatial derivatives are taken with respect to spatial coordinates. This particularity of the ALE formulation allows us to use a semi-discrete method.

#### Weak formulation of the cerebrospinal fluid flow problem

To solve the governing equations using finite elements, we first write the ALE description of the CSF flow problem in weak form. In our discretization, there is only one velocity field $\varvec{v}$ that is defined over the entire computational domain $\Omega$, and shared by the fluid and the solid. Because $\varvec{v}$ is a continuous field, the kinematic compatibility at the fluid-solid interface is satisfied without imposing any additional constraints. For simplicity and consistency, we also extend the pressure field to the entire domain $\Omega$, and we call *p* the extended field. To derive the weak form, we introduce the functional space $\varvec{{\mathscr {W}}}$, which is also defined over the entire domain $\Omega$. All components of the trial velocity $\varvec{v}$, the trial pressure *p*, as well as the test functions for velocity and pressure $\varvec{w}$ and *q* belong to $\varvec{{\mathscr {W}}}$.

Our weak form is based on a variational multiscale (VMS) formulation [57], which allows us to use the same functional space for all variables, and renders stable and accurate results without assumptions additional to those of the governing equations. The VMS formulation of the problem is: Find $\varvec{v}\in \varvec{{\mathscr {W}}}$, $p\in {\mathscr {W}}$ such that, for all $\varvec{w}\in \varvec{{\mathscr {W}}}$ and $q \in {\mathscr {W}}$,

26 $$\begin{aligned} {G}_f = {G}_{Gal} + {G}_{VMS} = 0 \qquad \end{aligned}$$

where

27 $$\begin{aligned} {G}_{Gal}&= \int _{\Omega ^f} \varvec{w} \cdot \rho ^f \frac{\partial \varvec{v}}{\partial t} \bigg |_{\widehat{\varvec{x}}} \, \text {d}v \nonumber \\&+ \int _{\Omega ^f} \varvec{w} \cdot \rho ^f (\varvec{v} - \widehat{\varvec{v}}) \cdot \nabla \varvec{v} \, \text {d}v + \int _{\Omega ^f} \nabla \varvec{w} : \varvec{\sigma }^f \, \text {d}v \nonumber \\&- \int _{\Gamma ^{m}} \varvec{w} \cdot \varvec{\sigma }^f \varvec{n}^f \, \text {d}a - \int _{\Omega ^f} \varvec{w} \cdot \varvec{f}^f \, \text {d}v - \int _{\Omega ^f} q \, \nabla \cdot \varvec{v} \, \text {d}v, \end{aligned}$$

and

28 $$\begin{aligned} {G}_{VMS} =&-\int _{\Omega ^f} \left( (\varvec{v} - \widehat{\varvec{v}}) \cdot \nabla \varvec{w} + \nabla q \frac{1}{\rho ^f} \right) \cdot \varvec{v}' \, \text {d}v \nonumber \\&- \int _{\Omega ^f} p' \cdot \nabla \cdot \varvec{w} \, \text {d}v \nonumber \\&+ \int _{\Omega ^f} \varvec{w} \cdot \varvec{v}' \cdot \nabla \varvec{v} \, \text {d}v - \int _{\Omega ^f} \nabla \varvec{w} : (\varvec{v}' \otimes \varvec{v}') \frac{1}{\rho ^f} \, \text {d}v. \end{aligned}$$

Here, $\varvec{v}' = -\tau _M \varvec{r}_M$ and $p' = -\tau _C r_C$, where $\varvec{r}_M$, $r_C$ are the residuals of the strong form of the coarse-scale momentum balance and mass conservation equations respectively. The stabilization parameters $\tau _M$ and $\tau _C$ are defined as

29 $$\begin{aligned} \tau _M&= \left( \frac{4}{\Delta t^2}+ (\varvec{v}-\widehat{\varvec{v}})\cdot \varvec{G}(\varvec{v}-\widehat{\varvec{v}}) + C_I (\nu ^f)^2 \varvec{G}:\varvec{G} \right) ^{-1/2},\end{aligned}$$

30 $$\begin{aligned} \tau _C&= \left( \tau _M \operatorname {tr}\varvec{G}\right) ^{-1}, \end{aligned}$$

where $\nu ^f=\mu ^f/\rho ^f$ is the kinematic viscosity, $\Delta t$ is the time step size, and $C_I$ is a positive constant, independent of the mesh size, derived from an inverse estimate. Here, we take $C_I=3$. The tensor $\varvec{G}$ is the element metric tensor obtained from the physical-parent element mapping. All the terms in Eq. (28) involve the residuals of the strong form equations, which become negligible for fine meshes. Hence, the overall algorithm retains higher-order consistency with the Navier–Stokes equations.

#### Weak form of the soft tissue mechanics problem

The weak form of the fluid problem was expressed in terms of trial and test functions defined on the entire computational domain $\Omega$. We will use the same trial velocity $\varvec{v}$ and test function $\varvec{w}$ to derive the weak form of the solid mechanics problems. This construction guarantees automatic satisfaction of the kinematic compatibility at the fluid-solid interface $\Gamma ^m$. The displacement field can be obtained from the velocity by integration of the equation $\varvec{v} = \partial _t \varvec{u} \mid _{\varvec{X}}$.

##### Epidural tissue

We derive the weak form of Eq. (3) by multiplying it with the test function $\varvec{w}\in \varvec{{\mathscr {W}}}$, integrating over the domain $\Omega ^s_0$ and integrating by parts. The problem is: Find $\varvec{v} \in \varvec{{\mathscr {W}}}$ such that for all $\varvec{w} \in \varvec{{\mathscr {W}}}$,

31 $$\begin{aligned}&G_{s} = \int _{\Omega _0} \varvec{w}\cdot \rho _0 \partial _t\varvec{v} \mid _{\varvec{X}} \text {d}V + \int _{\Omega ^s_0} \nabla _{\varvec{X}} \varvec{w} : \varvec{P}^s \text {d}V \nonumber \\&\quad - \int _{\Gamma ^m_0} \varvec{w} \cdot \varvec{P}^s \varvec{N}^s \text {d}A - \int _{\Omega ^s_0} \varvec{w} \cdot \varvec{f}_0^s \text {d}V = 0. \end{aligned}$$

where $\varvec{P}^s$ is the first Piola-Kirchhoff stress tensor of epidural tissue and $\varvec{N}^s$ denotes the outward facing unit normal vector to $\Gamma ^m_0$.

##### Dura mater

The weak form of the equations governing dura mechanics is: Find $\varvec{v} \in \varvec{{\mathscr {V}}}$ such that for all $\varvec{w} \in \varvec{{\mathscr {W}}}$,

32 $$\begin{aligned}&G_{m} = \int _{\Omega ^m_0} \varvec{w} \cdot \rho _0 \partial _t \varvec{v} \mid _{\varvec{X}} \text {d}V + \int _{\Omega ^m_0} \nabla _{\varvec{X}} \varvec{w} : \varvec{P}^m \text {d}V \nonumber \\&\quad - \int _{\Gamma ^m_0} \varvec{w} \cdot \varvec{P}^m \varvec{N}^m \text {d}A -\int _{\Omega ^m_0} \varvec{w} \cdot \varvec{f}_0^m \text {d}V = 0. \end{aligned}$$

where $\varvec{P}^m$ is the first Piola-Kirchhoff stress tensor of dura mater. In Eq. (32), integration occurs over the domain $\Omega _0^m \equiv \Gamma ^{m}_0 \times [0, \zeta ]$. Because $\zeta$ is small, we make the following approximations,

33 $$\begin{aligned} \int _{\Omega ^m_0} (\cdot ) \text {d}V = \zeta \int _{\Gamma ^{m}_0} (\cdot ) \text {d}A, \end{aligned}$$

34 $$\begin{aligned} \int _{\Gamma ^m_0} (\cdot ) \text {d}A = \zeta \int _{\partial \Gamma ^{m}_0} (\cdot ) \text {d}L. \end{aligned}$$

Using Eqs. (A10)-(A11), the weak form becomes

35 $$\begin{aligned}&G_{m} = \zeta \int _{\Gamma ^{m}_0} \nabla _{\varvec{X}}\varvec{w} : \varvec{P}^m \text {d}A - \zeta \int _{\partial \Gamma ^{m}_0}\varvec{w} \cdot \varvec{P}^m \varvec{N}^m \text {d}L \nonumber \\&\quad - \zeta \int _{\Gamma ^{m}_0} \varvec{w} \cdot \varvec{f}^m_0 \text {d}A. \end{aligned}$$

where we neglected the membrane’s inertia, as it scales with the membrane thickness and is negligible with respect to the inertia of the epidural tissue. In Eq. (35), $\varvec{f}^m_0$ denotes the body force on the membrane in the material configuration which is

36 $$\begin{aligned} \varvec{f}^m_0 = \frac{1}{\zeta }(\varvec{\sigma }^f\varvec{n}^f + \varvec{\sigma }^s\varvec{n}^s) \end{aligned}$$

The integral on $\partial \Gamma ^m_0$ in Eq. (35) vanishes because the displacement field is restricted on the line $\partial \Gamma ^m_0$.

#### Coupled mixed-dimensional FSI problem

The fluid and solid problems are coupled through kinematic and traction compatibility conditions. The kinematic compatibility conditions are satisfied automatically due to the use of a global velocity field. The traction balance condition can be expressed as

37 $$\begin{aligned} \varvec{\sigma }^f\varvec{n}^f + \varvec{\sigma }^s\varvec{n}^s = \varvec{0} \end{aligned}$$

because the membrane does not contribute to any out-of-plane stress. Using Eqns. (36, 37), summing all integrals on $\Gamma ^{m}_0$ in the combined weak form gives

38 $$\begin{aligned} \varvec{f}_0^m = \varvec{0} \qquad \text {on } \Gamma ^m_0, \end{aligned}$$

and

39 $$\begin{aligned} J\varvec{\sigma ^fF^{-T} N^f} + \varvec{P^s N^s} = \varvec{0} \qquad \text {on } \Gamma ^m_0, \end{aligned}$$

which is the pull-back of Eq. (37) to the reference configuration. Assuming that no body forces act on the fluid or the solid, the weak form of the combined problem including CSF flow and the mechanics of epidural tissue and dura mater is: Find $\varvec{v}\in \varvec{{\mathscr {W}}}$, $p \in {\mathscr {W}}$, such that, for all $\varvec{w} \in \varvec{{\mathscr {W}}}$, and for all $q \in {\mathscr {W}}$,

40 $$\begin{aligned} G_{MFSI}&= \int _{\Omega ^f} \varvec{w} \cdot \rho ^f \frac{\partial \varvec{v}}{\partial t} \bigg |_{\widehat{\varvec{x}}} \, \text {d}v \nonumber \\&+ \int _{\Omega ^f} \varvec{w} \cdot \rho ^f (\varvec{v} -\widehat{\varvec{v}}) \cdot \nabla \varvec{v} \, \text {d}v + \int _{\Omega ^f} \nabla \varvec{w} : \varvec{\sigma }^f \, \text {d}v \nonumber \\&- \int _{\Omega ^f} q \nabla \cdot \varvec{v} \, \text {d}v \nonumber \\&- \int _{\Omega ^f} \left( (\varvec{v} - \varvec{{\widehat{v}}}) \cdot \nabla \varvec{w} + \nabla q \frac{1}{\rho ^f} \right) \cdot \varvec{v'} \, \text {d}v \nonumber \\&- \int _{\Omega ^f} p' \cdot \nabla \cdot \varvec{w} \, \text {d}v \nonumber \\&+ \int _{\Omega ^f} \varvec{w} \cdot \varvec{v}' \cdot \nabla \varvec{v} \, \text {d}v - \int _{\Omega ^f} \nabla \varvec{w} : (\varvec{v}' \otimes \varvec{v}') \frac{1}{\rho ^f} \, \text {d}v\nonumber \\&+ \zeta \int _{\Gamma ^m_0} \nabla _{\varvec{X}} \varvec{w} : \varvec{P^m} \, \text {d}A \nonumber \\&- \int _{\Omega ^s_0} \varvec{w} \cdot \rho _0 \partial _t \varvec{v} \mid _{\varvec{X}} \, \text {d}V + \int _{\Omega ^s_0} \nabla _{\varvec{X}} \varvec{w} : \varvec{P^s} \, \text {d}V = 0. \end{aligned}$$

#### Mesh motion problem

The fluid mesh motion $\widehat{\varvec{u}}$ needs to satisfy two conditions: (i) it is compatible with the solid displacement at the fluid-solid interface, (ii) it provides a mesh of sufficient quality to compute the solution to the fluid problem. As long as these two conditions are satisfied, the fluid mesh motion is arbitrary. Here, we compute the fluid mesh motion by solving an elasticity problem, subject to time-dependent Dirichlet boundary conditions that impose compatibility with the displacement of the fluid-solid interface [58]. To avoid excessive distorsion of the fluid elements, we employ Jacobian-based stiffening, which makes more rigid the elements with higher distortion [59].

#### Spatial discretization

We utilized gmsh [60] to discretize the domain $\Omega$ using tetrahedra. The mesh is conformal at the fluid-solid interface. The size of the fluid elements that are near the solid walls is 5 times smaller than in the fluid interior to accurately resolve the boundary layers. We constructed functional spaces over the tetrahedral mesh using linear Lagrange polynomials. We use the parametrization of the finite element shape functions to describe the membrane mapping. The mesh has approximately 4.3 M and 2.7 M elements in the fluid and solid domains, respectively. This mesh resolution was chosen after a mesh refinement study. We tested coarser meshes with 3.2 M and 2.3 M elements (fluid and solid combined), and observed no notable differences in the flow and displacement fields.

#### Time integration and numerical implementation

Our time integration is based on the backward Euler method. We performed a time refinement study with time step sizes of $\Delta t = 0.625$ ms and $\Delta t = 0.25$ ms, and observed no notable differences in the numerical solution compared to $\Delta t = 1.25$ ms. We therefore used $\Delta t = 1.25$ ms as our time step size.

We adopt a quasi-direct solution strategy [57], where the mesh motion problem is solved separately from the coupled fluid-tissue problem. The fluid-solid problem and the mesh motion problem are nonlinear, therefore the form (40) is linearized using Newton–Raphson. We solve linear algebraic problems using the GMRES iterative solver with algebraic multigrid (AMG) preconditioner. The algorithm was implemented in the open-source finite element package FEniCS [61, 62].
