# Supplementary material for: Mixed-dimensional fluid–structure interaction simulations reveal key mechanisms of cerebrospinal fluid dynamics in the spinal canal
Source: Fluids Barriers CNS. 2025 Jul 30;22:81. doi: 10.1186/s12987-025-00691-4 (PMC12312592; doi:10.1186/s12987-025-00691-4)
Supplement: Supplementary file 1 — Supplementary material 1. [file 12987_2025_691_MOESM1_ESM.pdf]

Supplemental material: Mixed-dimensional fluid-structure interaction simulations reveal key mechanisms of cerebrospinal fluid dynamics in the spinal canal

Deshik Reddy Putluru[1], Adrian Buganza Tepole[1,2], Hector Gomez[1,2*]

[1*]School of Mechanical Engineering, Purdue University, 585 Purdue Mall, West Lafayette, 47906, Indiana, USA.
[2]Weldon School of Biomedical Engineering, Purdue University, 206 S Martin Jischke Dr, West Lafayette, 47906, Indiana, USA.

*Corresponding author(s). E-mail(s): hectorgomez@purdue.edu;

1 Parametric study of boundary conditions on the outer surface of epidural fat

Our baseline anatomical model assumes a uniform epidural fat layer with a constant thickness of 4 mm. However, the thickness of epidural fat in humans is highly non-uniform varying from 2 to 20 mm, and is surrounded by the vertebral bones [1]. One way to represent the surrounding vertebrae is to restrict the motion of outer wall of the epidural fat. However, because we adopt the physiological lower-bound thickness of 4 mm, such a restriction could artificially stiffen the subarachnoid space (SAS). An alternative approach is to impose the Robin boundary condition $\boldsymbol{\sigma}^s \boldsymbol{n}^s = -k\boldsymbol{u} - p_0 \boldsymbol{n}^s$, where k is parameter that represent the effective material behavior of tissues that surround the current anatomical structure of epidural fat and p_0 is pressure exerted by CSF and outer tissues.

In the absence of data for k, we conduct a parametric study by estimating plausible values based on two assumptions: i) the outer boundary of the anatomical model is surrounded solely by fat tissue, and ii) the mechanical response of the surrounding fat is linear. Therefore, we model the elastic support as $k = \frac{E}{t_{\text{ef}}}$, where E and t_{ef} are, respectively, the Young's modulus and thickness of epidural fat. To evaluate the

impact of k, we perform three simulations using thickness values of 5 mm, 10 mm, and 20 mm, which correspond to $k = 2 \times 10^6$ Pa/m, 1×10^6 Pa/m, and 5×10^5 Pa/m, respectively and analyze the effects of k on flow patterns and pressure pulsations.

1.1 Flow patterns

In Fig. 1, we compare the flow rate waveforms predicted by the FSI simulations performed with different values of k with the in-vivo data at C2-C3, C7-C8, T10-T11 locations. The simulations with non-zero values of k (see Fig. 1b, 1c and 1d), predict more than two flow reversals per cardiac cycle at C7-C8 and T10-T11 locations. This is in contrast with the in vivo data and our simulations for $k = 0$, which show exactly two flow reversal points per cardiac cycle. This suggests a larger number of pulse wave reflections from the caudal end, likely due to an excessively stiff SAS. This also results in qualitative disagreement of flow direction during the systolic phase, where the simulations predict caudal flow whereas the in vivo data and our simulation with $k = 0$ show strong cranial flow.

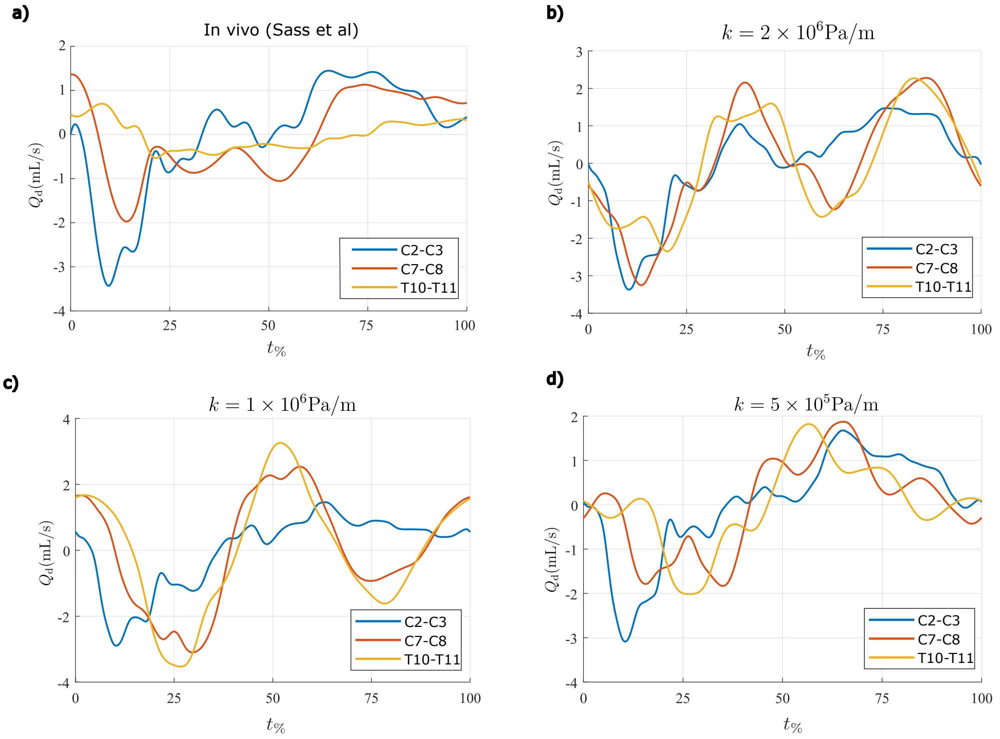

Fig. 1 Comparison of the flow rate waveforms from a) in-vivo data [2] with those predicted by the FSI simulations performed with b) $k = 2 \times 10^6$ Pa/m, c) 1×10^6 Pa/m and, d) 5×10^5 Pa/m at C2-C3, C7-C8, and T10-T11 levels.

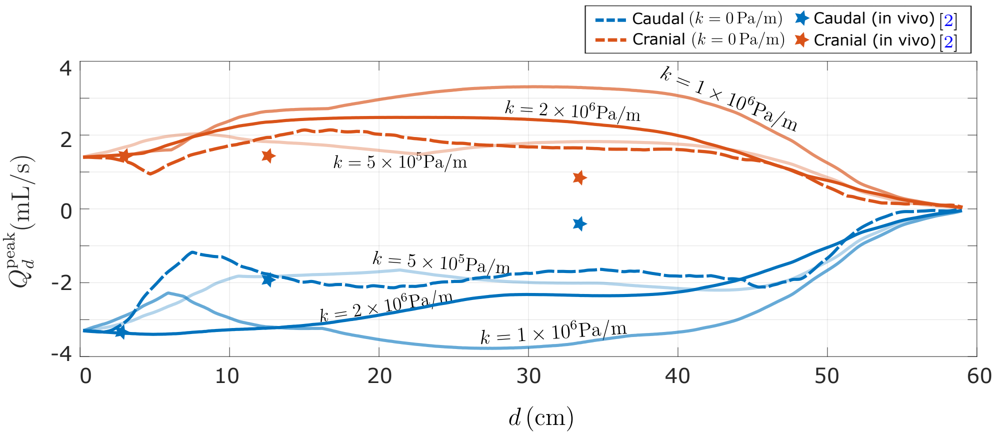

Fig. 2 Caudal (red) and cranial (blue) peak flow rates. FSI simulations with non-zero values of k (solid lines), FSI simulation with $k = 0$ (dashed lines) and in vivo measurement from [2] (stars).

To analyze the effect of k on craniocaudal decay of flow rates, we plot the peak CSF flow rate in cranial (red) and caudal (blue) directions in Fig. 2. The results from our FSI simulations with non-zero values of k are shown with solid lines, the results of FSI simulations with $k = 0$ are shown with dashed lines and the stars denote in vivo data [2]. The peak flow rates obtained from simulations with $k = 0$ and $k = 5 \times 10^5$ Pa/m show better agreement with in vivo data than those using $k = 1 \times 10^6$ Pa/m and 2×10^6 Pa/m.

1.2 Intraspinal pressure

We analyze the variations of intraspinal pressure pulsations in our simulations with different values of k. Fig. 3 shows spatio-temporal distributions of average pressure over a slice with respect to distance from foramen magnum and time over a cardiac cycle. We compare the simulation results with the measurements in the lumbar region from [3], which were obtained using a catheter. In our simulations with $k = 2 \times 10^6$ Pa/m and 1×10^6 Pa/m, the pressure pulsation amplitudes at the L2 level exceed 150 Pa, which is significantly higher than the clinical range of 4-10 mm H_2O (39-98 Pa). The amplitude of pressure waveform at L2 level in the simulation with $k = 5 \times 10^5$ is approximately 75 Pa which is within the clinical range. For reference, the pulsation amplitude at L2 for $k = 0$ is 35 Pa.

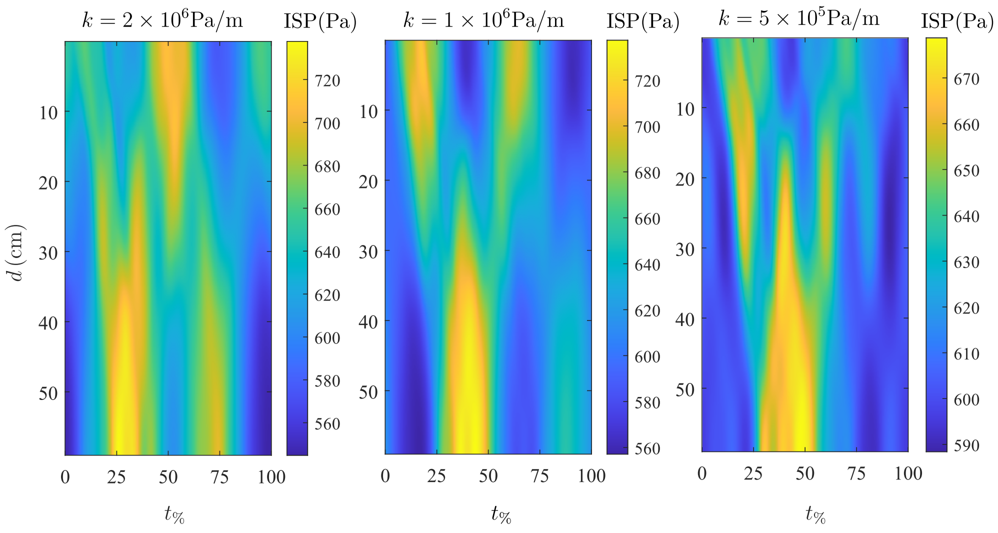

Fig. 3 Spatio-temporal distribution of absolute pressure for $k = 2 \times 10^6$ Pa/m, (left) 1×10^6 Pa/m, (center) and 5×10^5 Pa/m (right). The pressure ranges are higher when k is large and lower and agrees with clinical range when $k = 5 \times 10^5$ Pa/m.

1.3 Summary

Table 1 shows the level of agreement between the FSI simulations for different values of k and the in vivo data. Our simulation without elastic support ($k = 0$) shows the best overall agreement with in vivo data. While more accurate characterization of k—such as incorporating subject-specific epidural fat thickness—could potentially improve the agreement of simulated flow patterns and pressures, such data are currently sparse and not well established in the literature. Future studies could address this gap by integrating more detailed anatomical information as they become available.

Quantity	$k = 2 \times 10^6$ Pa/m	$k = 1 \times 10^6$ Pa/m	$k = 5 \times 10^5$ Pa/m	$k = 0$ Pa/m
Flow waveforms (qualitative)	Poor	Poor	Poor	Good
Pressure amplitude	Poor	Poor	Good	Good

Table 1 Level of agreement between simulation results for various values of k and in vivo data [2].

References

[1] De Andrés, J., Reina, M.A., Machés, F., De Sola, R.G., Oliva, A., Prats-Galino, A.: Epidural fat: considerations for minimally invasive spinal injection and surgical therapies. J Neurosurg Rev **1**, 45–53 (2011)

[2] Sass, L.R., Khani, M., Natividad, G.C., Tubbs, R.S., Baledent, O., Martin, B.A.: A 3D subject-specific model of the spinal subarachnoid space with anatomically realistic ventral and dorsal spinal cord nerve rootlets. Fluids and Barriers of the CNS **14**, 36 (2017) https://doi.org/10.1186/s12987-017-0085-y

[3] Tumani, H., Petereit, H., Gerritzen, A., Gross, C., Huss, A., Isenmann, S., Jesse, S., Khalil, M., Lewczuk, P., Lewerenz, J., *et al.*: S1 guidelines "lumbar puncture and cerebrospinal fluid analysis"(abridged and translated version). Neurological Research and Practice **2**, 1–28 (2020) https://doi.org/10.1186/s42466-020-0051-z
